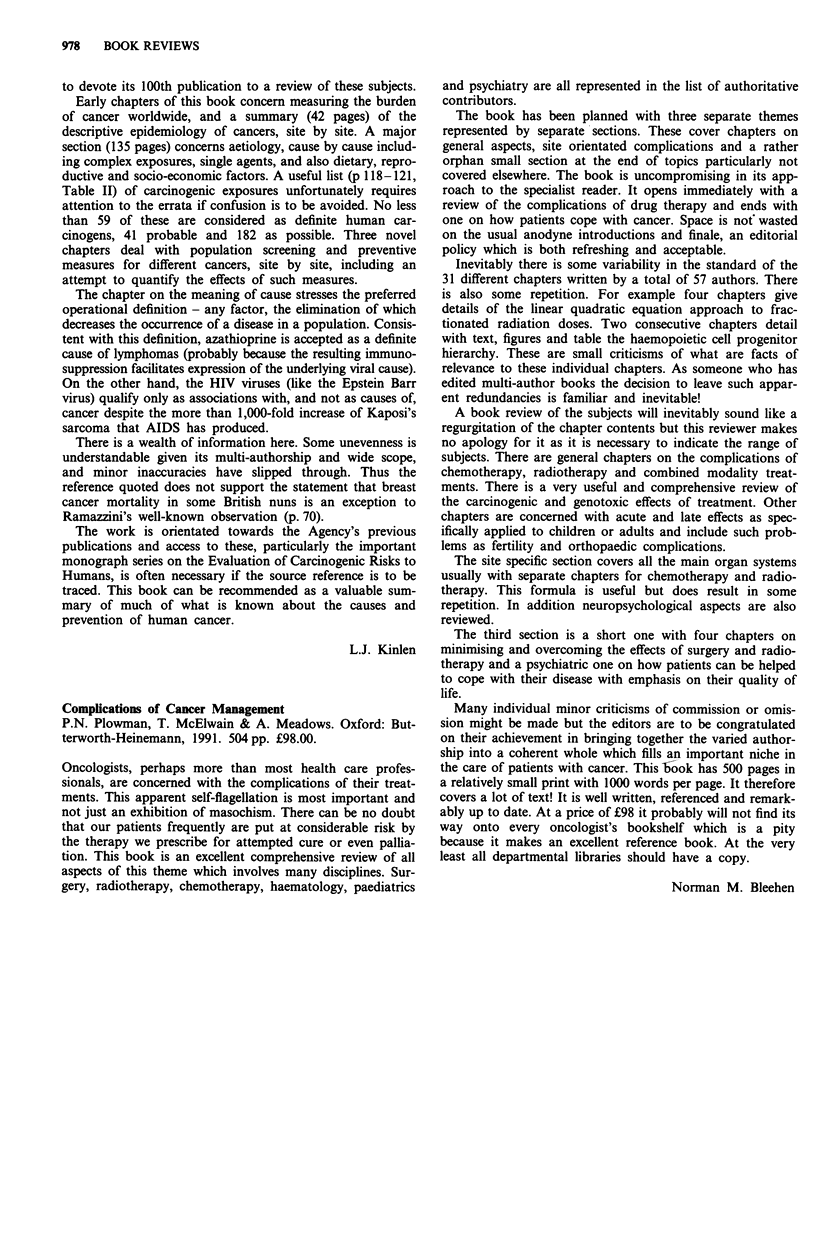# Complications of Cancer Management

**Published:** 1991-11

**Authors:** Norman M. Bleehen


**Complications of Cancer Management**

P.N. Plowman, T. McElwain & A. Meadows. Oxford: Butterworth-Heinemann, 1991. 504 pp. £98.00.

Oncologists, perhaps more than most health care professionals, are concerned with the complications of their treatments. This apparent self-flagellation is most important and not just an exhibition of masochism. There can be no doubt that our patients frequently are put at considerable risk by the therapy we prescribe for attempted cure or even palliation. This book is an excellent comprehensive review of all aspects of this theme which involves many disciplines. Surgery, radiotherapy, chemotherapy, haematology, paediatrics and psychiatry are all represented in the list of authoritative contributors.

The book has been planned with three separate themes represented by separate sections. These cover chapters on general aspects, site orientated complications and a rather orphan small section at the end of topics particularly not covered elsewhere. The book is uncompromising in its approach to the specialist reader. It opens immediately with a review of the complications of drug therapy and ends with one on how patients cope with cancer. Space is not wasted on the usual anodyne introductions and finale, an editorial policy which is both refreshing and acceptable.

Inevitably there is some variability in the standard of the 31 different chapters written by a total of 57 authors. There is also some repetition. For example four chapters give details of the linear quadratic equation approach to fractionated radiation doses. Two consecutive chapters detail with text, figures and table the haemopoietic cell progenitor hierarchy. These are small criticisms of what are facts of relevance to these individual chapters. As someone who has edited multi-author books the decision to leave such apparent redundancies is familiar and inevitable!

A book review of the subjects will inevitably sound like a regurgitation of the chapter contents but this reviewer makes no apology for it as it is necessary to indicate the range of subjects. There are general chapters on the complications of chemotherapy, radiotherapy and combined modality treatments. There is a very useful and comprehensive review of the carcinogenic and genotoxic effects of treatment. Other chapters are concerned with acute and late effects as specifically applied to children or adults and include such problems as fertility and orthopaedic complications.

The site specific section covers all the main organ systems usually with separate chapters for chemotherapy and radiotherapy. This formula is useful but does result in some repetition. In addition neuropsychological aspects are also reviewed.

The third section is a short one with four chapters on minimising and overcoming the effects of surgery and radiotherapy and a psychiatric one on how patients can be helped to cope with their disease with emphasis on their quality of life.

Many individual minor criticisms of commission or omission might be made but the editors are to be congratulated on their achievement in bringing together the varied authorship into a coherent whole which fills an important niche in the care of patients with cancer. This book has 500 pages in a relatively small print with 1000 words per page. It therefore covers a lot of text! It is well written, referenced and remarkably up to date. At a price of £98 it probably will not find its way onto every oncologist's bookshelf which is a pity because it makes an excellent reference book. At the very least all departmental libraries should have a copy.

Norman M. Bleehen